# A Real-World Dataset for detecting Handwashing in daily Life using Wrist Motion Data from Wearables

**DOI:** 10.1038/s41597-026-06698-2

**Published:** 2026-02-05

**Authors:** Robin Burchard, Kristina Kirsten, Marcel Miché, Philipp Scholl, Bert Arnrich, Kristof Van Laerhoven, Roselind Lieb, Karina Wahl

**Affiliations:** 1 University of Siegen, Siegen, Germany; 2 Hasso Plattner Institute, Potsdam, Germany; 3 University of Freiburg, Freiburg, Germany; 4 University of Basel, Basel, Switzerland

**Keywords:** Therapeutics, Psychology

## Abstract

Handwashing detection is a relevant research topic with applications in healthcare and professional environments. While usually related to hygiene improvement, handwashing detection could also be used to support individuals with obsessive-compulsive disorder (OCD). For these individuals, compulsive, long, and frequent handwashing has a negative impact. An automated system could spot compulsive handwashing in real-time and augment the therapy process. No activity recognition datasets containing in-the-wild-recorded compulsive handwashing are available. With this work, we present the OCDetect Dataset, the first dataset with unscripted, compulsive handwashing. It contains recordings from inertial measurement units (IMUs) of 22 participants over 28 days, with 3000 recorded hand washes. For each hand wash, we supply its user-annotated kind (compulsive / routine). We provide an overview of related datasets and describe the recording, cleaning, labeling, and final features of our dataset. We reach a maximum F1 score of 0.77 (avg.: 0.33, chance level: 0.03) when spotting handwashing from all background activities on unseen participants. Our dataset and code for the reproduction of our results are publicly available.

## Background & Summary

Handwashing is an essential part of personal hygiene because it can reduce the amount of pathogens on our hands significantly^1^. For certain professions or activities, frequent and effective handwashing is an absolute must. Examples of this include but are not limited to hospital workers and other medical professionals, laboratory staff, or workers in the food industry. Added to that, handwashing is a crucial part of every human’s personal hygiene. Appropriate handwashing leads to significant benefits in terms of reducing the incidence of infection^2^. Therefore, improving the quality of hand washes is a desirable aim in general, which can also be supported by modern technology. Wearable and camera-based handwashing reminder and quality rating systems have been proposed in the past. It is usually claimed that automatic handwashing detection and rating systems could make sure that handwashing is done frequently and thoroughly enough and with the correct sequence of steps. However, a handwashing detection system’s use case is usually limited to a relatively specific application scenario, for which the system was developed. Training data for a general handwashing detection model that is supposed to detect handwashing in everyday life is rarely available. For training such a model, a sufficient number of handwashing examples is needed. Although research has been conducted in this field, there are only a few small public datasets containing realistic instances of everyday handwashing.

Another, less-researched, aspect of handwashing can be observed in individuals with obsessive-compulsive disorder (OCD). Individuals with OCD frequently experience excessive washing compulsions, typically characterized by prolonged and/or extremely frequent episodes of ritualized handwashing. Individuals affected by OCD experience unwanted intrusive thoughts. For example, the fear of being contaminated by touching a brown spot on the floor. Although often recognized as being exaggerated or irrational, these thoughts typically lead to great distress and the urge to act to prevent something terrible from happening (compulsive behaviors)^3^. The different sub-types of OCD that can be distinguished range from ordering symmetry concerns to checking behaviors, over taboo thoughts, and to serious contamination concerns and cleanliness/washing behaviors. Typically, in individuals with the latter symptoms, distressing contamination fears result in excessive cleanliness and washing behaviors, including excessive handwashing. Washing compulsions, including handwashing, are very common in individuals with OCD (47.2 %-67.7 %)^4^ and impairing. OCD often harms the quality of life of the affected individuals and their families^5^. The distinction between routine and compulsive hand washes is not always obvious, as the subjective perception of a hand wash can be different from a rational and objective observation. Obsessive-compulsive handwashing can be characterized by repetition of rituals, handwashing frequency, and often by a longer than average washing duration. Added to the mental aspect, the overly frequent and intensive washing could lead to skin diseases such as contact dermatitis, especially when done with antibacterial soap^6^. OCD is treated with cognitive behavioral therapy (CBT), which includes exposure and response prevention^7–10^. Exposure and response prevention means that, in therapy sessions (at the therapist’s office or at home), the individual with OCD is exposed to a feared stimulus or situation, that activates their obsession and is simultaneously prevented from performing the compulsive action.

Automatically detecting the onset of compulsive handwashing could help patients refrain from washing and instead engage in a therapeutically beneficial activity. For example, if a smartwatch alerts the patient that they are about to wash compulsively (e.g., via a vibration), they could choose to stop washing and instead perform an exposure exercise by touching a feared stimulus. To the best of our knowledge, no such system for detecting real-world compulsive handwashing currently exists. Aiming to improve both handwashing detection and the automatic distinction between compulsive and routine handwashing, we created and published the OCDetect dataset, which contains 2,930 instances of routine and obsessive-compulsive handwashing. The dataset consists of 2600 total hours of all-day recordings from a wrist-worn inertial measurement unit (IMU) integrated into a smartwatch. Our dataset is freely available to other researchers and is expected to reduce the data collection effort required for future studies related to handwashing detection.One of the motivations for creating this dataset is the potential to leverage machine learning technology in wearable devices, such as smartwatches, to support therapy for individuals with OCD^11^. Such technology could be applied in multiple ways to the assessment and treatment of OCD. For example, real-time, objective behavioral data gathered by a smartwatch could complement questionnaire data. By automatically detecting both routine and compulsive handwashing and requesting user feedback, a journal could be created to track progress. In the next step, just-in-time interventions, such as vibrations or sound signals triggered by the detection of compulsive washing, could remind the individual to perform response prevention exercises. Such a system would need to operate in real time with high specificity and sensitivity, remaining unobtrusive while avoiding missed intervention opportunities. An optimal system would integrate seamlessly with established treatment processes, be prescribed by the treating psychologist, and support therapy. During the prescription period, it could be further adapted to the user’s specific handwashing patterns using personalization methods.

Multiple sensing modalities can be used to detect handwashing, with the most common being camera-based and motion-sensor-based methods. For handwashing, which often occurs in bathrooms, vision-based approaches are suboptimal due to privacy concerns. They also require either a stationary camera at every sink or a body-worn camera. In contrast, wrist-worn sensors are readily available in modern smartwatches due to their integrated IMUs. Such devices can be worn anywhere without installation at specific sinks, and their wrist placement allows them to capture handwashing-specific movements. For these reasons, we chose wrist-worn smartwatches for our dataset. The difficulty of classifying activities of daily living (ADL) from motion-sensor data depends not only on the ambiguity of the movements but also on the duration of the activity. Longer activities are generally easier to detect in a continuous data stream. In a full day of typical activities, handwashing constitutes only a small fraction of the time. In real-world scenarios, we aim to detect these short events reliably, avoiding both false positives and missed instances. A realistic training and test set should therefore include as many background activities as possible. Furthermore, to approximate real-world performance, the target class of handwashing should not be overrepresented in the validation or test data. We therefore opted for all-day recordings, capturing the naturally occurring number of handwashing events without artificially altering the class balance.

### Objectives

Our study was guided by objectives aimed at creating a dataset that closely resembles real-world scenarios. Key elements of our approach include:

- **Developing a dataset that reflects real-world conditions** by not arbitrarily excluding cases of potentially invalid data, thereby accurately capturing the variability that occurs in practice.
- **Ensuring participant diversity** to encompass a wide range of scenarios and circumstances.
- **Utilizing a minimal technical setup** to maintain realism and participant commitment, emphasizing unobtrusiveness.
- **Integrating both objective measurements**, such as motion data, and **subjective assessments** through questionnaires to gain a comprehensive understanding of the collected data.
- **Evaluating the feasibility** of our approach for future studies and measuring participant acceptability of the setup to provide a foundation for subsequent research efforts.

### Related Work

Handwashing detection is an active research area with approaches for both general and compulsive handwashing, and published work exists for each, including datasets.

#### Handwashing Detection

Research on handwashing detection can be grouped into vision based and sensor based approaches. These include direct or camera based observation, real time locating systems, and sensor assisted observation^12^. In addition to IMU based sensing, moisture or grounding based detection is feasible using a wrist worn worn Electromyography (EMG)^13^, capacitive sensing^14^, a smart ring^15,16^, or air humidity sensors^17^. As argued in the previous section, body-worn sensing systems without cameras offer advantages in privacy and availability. Nevertheless, vision-based handwashing detection and assessment have also been studied, for example by Lulla *et al*.^18^, Wang *et al*.^19^, and Khamis *et al*.^20^. Lulla *et al*. published their video dataset, which contains handwashing procedures according to the World Health Organization (WHO)’s guidelines. Notably, the Apple Watch can remind users to wash their hands and detect handwashing based on movement and microphone data^21^. However, Apple’s technology is proprietary, and its methods remain unpublished. Most research on handwashing detection using sensors focuses on assessing handwashing quality, evaluating its frequency, or addressing both aspects simultaneously. Some proposed systems, for example, are used in intensive care units (ICUs), where staff can be reminded to wash their hands before entering the unit. There are many other professions in which high-quality and frequent handwashing is essential for sanitary reasons.

##### Existing Research

In a study by Mondol *et al*.^22^, participants wore smartwatches on their wrists, and a Bluetooth beacon was installed at a sink so that the handwashing detection model only ran when the participant was near the designated sink. They collected and annotated ground truth data, which, to our knowledge, was not made publicly available. Mondol *et al*. also introduced the HAWAD dataset^23^, used to evaluate their method for out-of-distribution detection of unseen sensor samples. Cao *et al*. employed a CNN-LSTM network similar to DeepConvLSTM^24^ to distinguish gestures related to handwashing and recorded a small dataset for their study, which was not available online^25^. In a recent study, Zhuang *et al*.^26^ combined microphone and IMU data to detect disinfection, face touching, and handwashing. Their dataset, to our knowledge, was also not made publicly available, and publishing microphone data from all-day recordings raises privacy concerns. Li *et al*.^27^ used a Naive Bayes classifier combined with Hidden Markov Models to automate the assessment of handwashing routines and detect steps according to WHO guidelines. Their data was recorded in-lab and also not publicly released.

##### Available Datasets

An overview of available datasets containing handwashing activities is provided in Table 1. Mallol-Ragolta *et al*.^28^ introduced the harAGE dataset, which includes approximately 53 minutes of accelerometer data for handwashing from 18 participants. Participants simulated handwashing motions without running water for about three minutes each. Wang *et al*.^29^ recorded 20 participants rubbing and washing their hands according to WHO guidelines to detect individual steps and assess washing quality. Their dataset is not publicly available but can be requested from the authors. Lattanzi *et al*.^30^ focused on detecting unstructured handwashing (that is, without a fixed gesture order) and collected their own dataset of handwashing, hand rubbing, and background activities ("other”) using accelerometer and gyroscope sensors. They combined this with the Daily Living Activities dataset (DLA^31^) and achieved an F1 score of 0.932 using a CNN-based model. Their dataset was shared with us upon request, but to our knowledge, has not been published. Zhang *et al*.^32^ recorded handwashing alongside selected ADL including sitting, typing, walking, ascending and descending stairs, and brushing teeth from ten participants, achieving an F1 score of 0.9871 for distinguishing handwashing from the other activities. Replication data for their work is available^33^. Most handwashing IMU data is collected in controlled settings with specific instructions rather than in-the-wild. To enable reproducibility and better estimate real-world performance, a public dataset of naturally occurring, non-influenced handwashing is desirable. The OCDetect dataset addresses this need.

**Table 1:** Comparison with other handwashing IMU datasets. All listed datasets use wrist or lower arm worn IMU sensors, and where duration was not reported, it was estimated from the authors’ description (participants  × repetitions  × duration per wash). Abbreviations: HW = handwashing, CHW = compulsive handwashing, ADL = activities of daily living, DA = dynamic activities (e.g., walking, running, sitting, stairs).

Dataset or Author	Contained activities	Type(s) of HW	Duration of HW	Public?
**OCDetect**	**All day ADL**	**Routine & compulsive**	**~31 h**	**yes**
harAGE^28^	7 DA & enacted HW	Enacted HW (w/o water)	~53 min	yes
Ablutomania^36^	ADL & enacted (C)HW	Enacted rout. & comp,	~6.5 h	yes
Zhang *et al*.^33^	ADLs & scripted HW	WHO HW steps	~40 min	yes
Wang *et al*.^29^	Hand rubbing & scripted HW	WHO HW steps	~11 h	no
HAWAD^23^	ADLs & scripted HW	WHO HW steps	~2.5 h	no

#### Obsessive-compulsive handwashing

For our additional research interest, compulsive handwashing, previous studies exist based on simulated compulsive handwashing. In a series of studies by Wahl *et al*. and Burchard *et al*.^11,34^,^35^, lab-recorded wrist-IMU data that was collected under controlled circumstances, was used to simulate compulsive handwashing. This was done in collaboration with scientists from the field of clinical psychology. The researchers used descriptions of handwashing and compulsive handwashing from individuals with OCD to create detailed scripts of compulsive handwashing, with a defined order of selected handwashing gestures. With this lab-recorded data, it was shown that enacted compulsive handwashing can be distinguished from routine handwashing of healthy participants. In another study, some other repetitive activities that were similar to handwashing were added to their dataset^36^. This was done to improve the robustness of the trained models against these activities. The confounding activities e.g. “rinsing a cup” contained repetitive wrist motion that was meant to be similar to handwashing. The researchers were able to show that it was possible to distinguish the enacted compulsive handwashing from the confounding activities including routine handwashing, with an accuracy of 0.823 and F1 score of 0.864. Their combined dataset is called “Ablutomania-Set” and is publicly available. However, to the best of our knowledge, no study has been published that includes handwashing sensor data from individuals diagnosed with OCD. Therefore, the dataset presented in this paper is the first dataset with IMU handwashing recordings collected from individuals with OCD in-the-wild.

## Methods

**Fig. 1:**
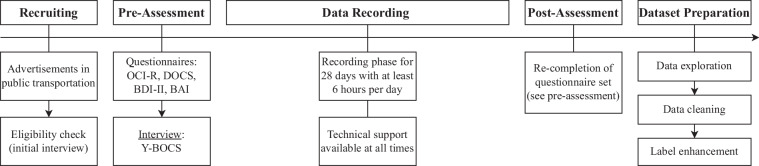
The procedure used for the creation of our dataset. The data recording took place during the four weeks between the pre-assessment and the post-assessment. The participants were instructed to report any technical difficulties they experienced to the researchers immediately. After the recording phase of all participants had ended, we started analyzing and preparing the dataset. Abbreviations: OCI-R: Obsessive-Compulsive Inventory, Revised^39^, DOCS: Dimensional Obsessive-Compulsive Scale^40^, BDI-II: Beck Depression Inventory-II^37^, BAI: Beck Anxiety Inventory^41^, Y-BOCS: Yale-Brown Obsessive Compulsive Scale^42^.

To collect realistic data, including handwashing activities, real-world daily life data from participants had to be collected. To achieve a sufficiently wide range of activities and individuals, we aimed for 15 to 20 participants. The participants were asked to wear a smartwatch for at least six hours a day over a 28-day recording period. The Android-based smartwatches were programmed to start a recording once they were removed from their charging bay. The recording would then continue over the day and automatically be stopped once the participant placed the watch back onto the charging bay, usually in the evening. Due to the all-day recordings, we were able to collect a lot of unlabeled background activity, so-called NULL data, that realistically represents the participant’s activities of daily living without specific information about its kind. Possibly confounding activities of the same user can therefore be part of a training set for a machine learning algorithm, which will likely make it easier to personalize the handwashing detection models. The entire process is illustrated in Fig. 1 and will be explained in more detail in the following sections.

### Recruitment and Participant Selection

A total of N = 30 participants were recruited from an outpatient clinic specializing in the treatment of OCD and through advertisements in public transportation in Basel, Switzerland. Eight participants dropped out before completing the four-week study due to personal reasons or technical issues, and their data were therefore considered insufficient or unreliable for analysis. Reported technical problems included limited battery life, which led to data loss when participants were unable to recharge the device during the day. In one case, flight mode was accidentally activated, preventing the recording of daily evaluations. The high volume of data processing caused the device to slow down or become unresponsive at times, resulting in participant frustration and, in some cases, discontinuation of use. One participant withdrew from the study because confirming handwashing events at work was not feasible. In three cases, the device became unusable and had to be replaced. All participants signed informed consent forms and agreed to the anonymous publishing of the recorded data. The Ethics Committee of North/West Switzerland (application number 2021-01317) approved the study. Our study complies with the declaration of Helsinki. A total of N=22 participants successfully completed the study. All participants completed a questionnaire set during an initial interview with the psychologists. To be included in this study, participants had to be aged between 18 and 75, non-suicidal (according to the BDI-II^37^, explained below), and meet the criteria for compulsive handwashing according to the DSM-5^38^ criteria of compulsions. Compulsions are defined by (1), (2):

1. Repetitive behaviors (handwashing) that the individual feels driven to perform in response to an obsession or according to rules that must be applied rigidly.
2. The handwashing is aimed at preventing or reducing anxiety or distress or preventing some dreaded event or situation; however, handwashing is not connected in a realistic way with what it is designed to neutralize or prevent, or is clearly excessive.

The questionnaire set contained multiple standardized questionnaires, which were included in order to describe the sample group in terms of symptom severity and other clinical aspects. The following questionnaires were used:

- the Obsessive-Compulsive Inventory, Revised (OCI-R,^39^, to assess distress caused by OCD symptoms (washing, checking, ordering, obsessing, and neutralizing) Version 01, 15.06.2021 8/43
- the Dimensional Obsessive-Compulsive Scale (DOCS,^40^) to assess avoidance, anxiety, resistance, and impairment caused by OCD symptoms (contamination, responsibility for harm, unacceptable thoughts, and symmetry)
- the Beck Depression Inventory-II (BDI-II^37^) to assess depressive symptoms
- the Beck Anxiety Inventory (BAI,^41^) to assess anxiety symptoms

Additionally, the severity of OCD symptoms was rated with a standardized interview, the Yale-Brown Obsessive Compulsive Scale (Y-BOCS)^42^, by trained researchers. Self-reported acute suicidal intent according to item nine of the BDI-II was an exclusion criterion for the study.

The participants’ mean age was 30.9, with a standard deviation of 11.55. The youngest participant was 18 and the oldest was 56 years old. 15 participants were female and seven were male. The mean score of the Y-BOCS scale (possible values: 0-40) among the participants was 15.48 ± 8.94, with a range from 3 to 30. Values under 7 are likely connected to sub-clinical symptoms, and 8-15 indicate a mild case of OCD. Values in the range of 16-23 are a sign of a moderate case of OCD, while 24-31 stipulate a severe case of OCD, and 32-40 relate to an extreme case of OCD. Seven out of the 22 participants were undergoing treatment for OCD while we conducted the study, while the remaining 15 were not.

### Technological Setup

We supplied each participant with an Android-based smartwatch (TicWatch 3 Pro LTE or TicWatch S) to wear during the study period. The smartwatches were adapted so that unnecessary applications were uninstalled and only the essential functions were retained. A special application was then installed that starts recording as soon as the device is removed from the charger. We recorded the in-built accelerometer and gyroscope at 50 *Hz*. The smartwatches were connected to the internet via a cellular WiFi hotspot, which was also provided to the participants free of charge. Once a recording was completed, the recording was uploaded to a web server via an encrypted connection. The password-protected web server then made the recordings available to the researchers.

The participants were asked to press an on-screen button immediately after they washed their hands during the day. This was done to gather label information for the dataset. To support the participants in remembering to label their hand washes, a DeepConvLSTM-based^24^ model was also employed to run directly on the smartwatch to detect hand washes and prompt the user for confirmation, once a detection was made. However, the model was trained on publicly available lab-collected handwashing data from various other participants^36^. While conducting the 28-day study with multiple participants, we found that the pre-trained model did not generalize well to new participants, and the prompts were mostly declined. Participants reported that the watch frequently confused handwashing with other daily activities, including hygiene-related actions such as brushing teeth and changing diapers, household chores such as washing dishes by hand, hanging or taking down laundry, cleaning, and packing a suitcase, work-related tasks such as kneading dough in a bakery or clearing tables in a restaurant, and other arm-intensive movements such as motorcycling, playing cards, brushing hair, walking, and showing a ticket on public transport. In addition, we did not record participants’ handedness or track which wrist the watch was worn on, which may have further contributed to the reduced performance of the pre-trained model. Therefore, most labels were set by the users by pressing the dedicated on-screen button for “Just washed your hands?”. We did not include a user interaction (like a button press) *before* washing hands, in order not to influence the handwashing behavior of the users. According to the OCD expert’s experiences and concerns, any interaction that makes the user aware of being “watched” by the smartwatch could lead to reactivity effects, i.e., the handwashing would be affected by pressing a button before starting the wash. These reactivity effects could include a variety of changes in washing. For example, hands might be washed for longer than usual because the patient thinks that touching the smartwatch might have contaminated his / her fingers. Or the wash could be shorter than usual or not take place at all because the patient might have become more aware of his / her ritual and might therefore deliberately resist the urge to wash. In any case, reactivity would interfere with the aim of collecting sensor data on naturally occurring compulsive handwashing.

**Fig. 2:**
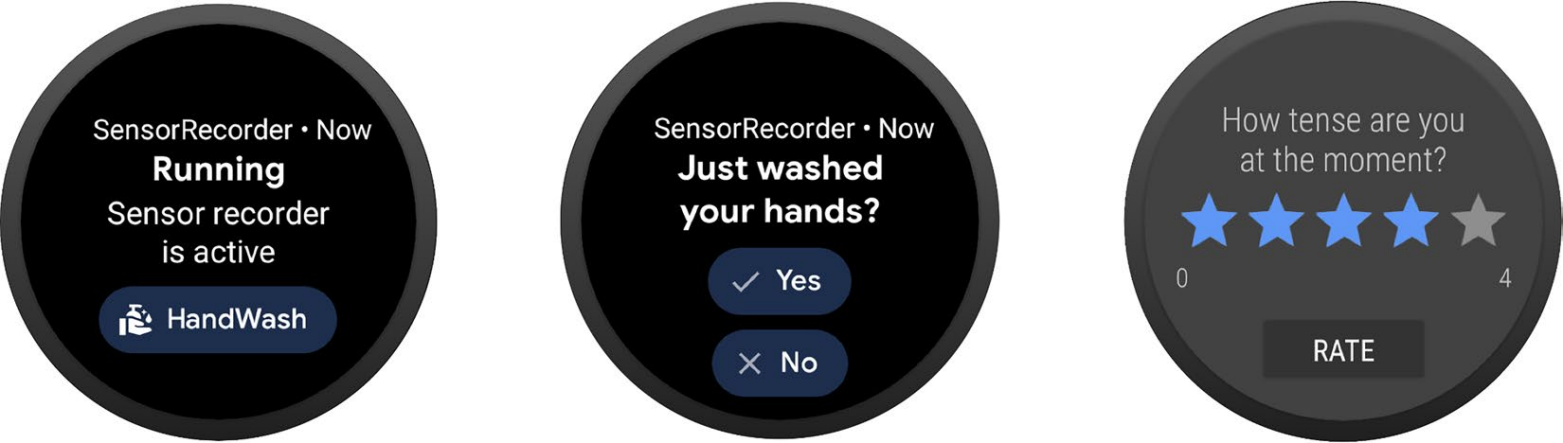
Interface displayed on the smartwatch. The leftmost screenshot shows the default state. The application shows the user that a recording is running, and the button to annotate a hand wash event is displayed. The center screenshot shows the question displayed to the user after an automatic detection of a hand wash. The user can either confirm the hand wash by pressing “Yes”, or correct the detection by pressing “No”. The screenshot on the right shows the rating scale used to measure the urge to wash hands and the current distress of the user. The user could select values from 1 to 5 on the scale by tapping or sliding on the stars and confirming by pressing the “Rate” button.

In addition to the yes/no question or the singular button press to confirm handwashing, the participants answered on-screen questions on the smartwatch for every hand wash. They were asked to classify the hand wash as “compulsive” or “not compulsive” (routine). The participant then rated the “urge” to wash their hands and their current “distress” on a scale from 1 to 5, with 1 being the lowest urge to wash hands or current distress and 5 the highest. Figure 2 displays the corresponding smartwatch user interface for several of the described interactions, while Table 2 shows the exact wording of the self-evaluation questionnaire.

**Table 2:** Questionnaire answered by the participants after each tagged hand wash.

**Question**	**Was this washing compulsive?**	**How strongly do you feel the urge to wash at the moment?**	**How tense are you at the moment?**
**Answers**	Yes / No	1–5	1–5

The smartwatch application also sent a daily notification at 6 p.m. asking the user to answer three additional questions about their self-assessment of the day. These questions were:

1. Overall, how frequent was your handwashing today?
2. Overall, how intense was your handwashing today?
3. Overall, how often did you confirm washing your hands today?

The participant could answer these questions on a scale from 1 to 5, similar to the prompts after confirming an instance of handwashing. The questions (1) and (2) were added to investigate the clinical utility of the sensor data. Question (3) was added to get an indication of the adherence to the instructions to confirm handwashing on the smartwatch.

### Dataset Aquisition

After the diagnostic interview in the laboratory, participants were provided with a smartwatch and instructed on how to use the on-screen buttons as well as the charging, recording, and uploading procedures. The participant then went to a specifically reserved bathroom, which contained a camera. In this bathroom, the participants conducted one example of handwashing, which was recorded by the soap-dispenser camera. By this, we made sure to have one clean and comprehensive handwashing activity for later analyses and comparison with handwashing in completely uncontrolled environments. Added to that, the videos also made a person-specific baseline hand wash duration available to us for each participant. The recorded videos are not part of the dataset and cannot be made publicly available for privacy and ethics-related reasons. After conducting the supervised instance of handwashing, the participants departed and the recording period of 28 days started. The individuals with OCD were asked to report any difficulties and problems directly to the researchers so that arising problems could be tackled immediately. The participants were asked to wear the smartwatch for at least six hours every day during the 4 week experiment period. After 28 days, the participants returned the smartwatch and were interviewed again by using the Y-BOCS test (see Fig. 1). The recordings were stopped, and the smartwatch was prepared for the next participant.

## Data Records

The dataset is available on Zenodo^43^. The provided files contain all information used in our experiments, split on a one-file-per-recording basis. One recording usually corresponds to one day of data for one participant, but the recordings can also be significantly shorter and multiple recordings from the same day can exist for each participant. The top-level folder contains two folders, “preprocessed” for all participants, “preprocessed_relabeled” for the six participants that were manually relabeled. All recording files can be attributed to the participants using the beginning of their file names (*OCDetect_id*). The file names also contain an incrementing number, starting at 0 for each participant, as well as a globally unique identifier (uuid-string) for each recording. The dataset also contains a file called “recording_metadata_table.csv”, which provides the date, time, and duration for each recording.

### File Format

Each recording file is a csv-file with the rows containing the recorded data, organized in a fixed list of columns. Each line of the file represents one data-point, recorded at 50 Hz.

#### General Columns

The first column, “timestamp”, contains the nanoseconds since the respective recording started. The next six columns relate to the three axes of the accelerometer and gyroscope, respectively. The other columns contain the raw user-label input, “user yes/no” containing all button presses by the user, in single lines. The columns “compulsive”, “tense”, and “urge” only contain data if the user confirmed a hand wash. These three columns hold the aforementioned user-annotation of the compulsiveness of the confirmed hand wash and the user’s self-rated tenseness and urge to wash the hands related to the washing event. As described in Section: Data Pre-processing, we include the “ignore” column, which contains 0 if the data should not be ignored and different non-zero values if the data is likely irrelevant (cf. Table 3). In the “relabeled” column, we store the automatically generated dense labels, 0 for NULL, 1 for routine, and 2 for compulsive hand wash.

**Table 3:** Data cleansing rules aimed at removing irrelevant and misleading data automatically from the dataset. 1. Rules for deleting entire files due to being invalid. 2. Rules for ignoring only parts of otherwise valid recordings. We either ignore regions of multiple seconds, minutes, or hours (2.1, 2.2) or only the labels therein (2.3-2.5).

id	Reason		Action	Description
1.1	File corrupt		Delete Recording	File empty or header only
1.2	Small duration			Too short to contain valid data
1.3	No movement			Too little movement to contain valid data
1.4	Wrong date			The recording precedes the experiment period
2.1	Initial Handwash		Ignore Region	First hand wash was conducted under supervision
2.2	Idleness			No movement over a certain period of time
2.3	Label early..	..in recording	Ignore Label	Invalid user label detected due to temporal context
2.4		..after idle		
2.5		..after label		

#### Columns For Relabeled Files

As we manually relabeled six of the participants (cf. Section: Participant-Specific Insights), we included additional columns in the processed recording files for these participants. The columns “annotator_1” and “annotator_2” contain the raw annotations by the two annotators who annotated each recording (cf. Table 5). The “merged annotation” column holds the combination of the annotator’s annotations, as described in Section: Manual Relabeling. The provided relabeled data used the “union” merging strategy. Regions with handwashing are indicated by the value 1 in the column “merged annotation”. Finally, the column “compulsive_relabeled” contains information on whether the manually annotated regions correspond to a routine or a compulsive hand wash (1 for compulsive and 0 otherwise).

## Technical Validation

To validate the collected and annotated dataset, we developed a complete pipeline for data preparation, calculated and visualized descriptive statistics, performed statistical tests, and carried out several machine learning experiments. The methodology and results of each step are described in detail in this section.

### Initial Validation and Processing

**Fig. 3:**
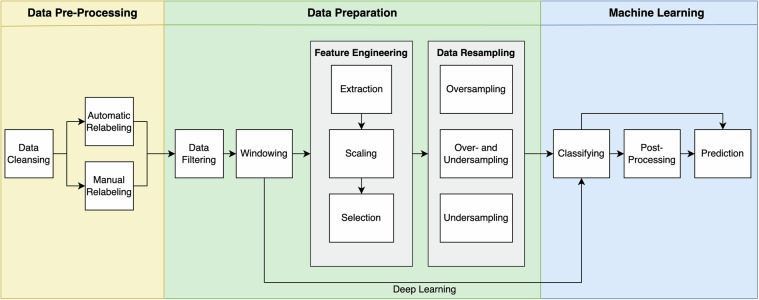
The complete pipeline for handling the dataset can be divided into three main stages: *Data Pre-Processing*, *Data Preparation*, and *Machine Learning*. This pipeline also demonstrates various options available, such as different relabeling or resampling strategies, tailored for different phases of the process.

To validate the compiled dataset, we developed a comprehensive, customizable, and flexible pipeline, shown in Fig. 3. The three main stages, *Data Pre-Processing, Data Preparation*, and *Machine Learning*, are described in the following subsections, and initial classification results are presented in Section Evaluation.

#### Data Pre-processing

The recorded data was analyzed and cleaned before it was exported. During both manual and automatic exploration of the recorded data, we realized that some parts of the all-day recordings were not useful, e.g., because some recordings did not contain any movement. We defined a set of rules based on which we objectively decide to ignore or remove data that we consider not to be useful from the dataset. The rules were derived together with the expert psychologists and are explained below.

##### Data Cleansing

We split the objective rules used for the cleaning of the data into two categories: (1) Rules that delete the entire recording, (2) Rules that mark parts of a recording file as invalid. We chose not to directly delete data from otherwise useful recordings but rather to mark them as to be ignored in order to preserve the spacing and order of the samples and so that other researchers who might disagree with our reasoning could, for example, still have access to idle periods in the recordings. The exact rules we used to cleanse the data are listed in Table 3.

To define the term “movement” in this context, we start by sliding a 500-sample long window (10s) over the accelerometer data with an overlap of 50 % and calculate the vector magnitude $\sqrt{{x}^{2}+{y}^{2}+{z}^{2}}$ at each data point. For each window, we compare the standard deviation of the window’s magnitude values to a threshold value of 0.2 *m*/*s*^2^. By doing this and applying the rules 1.3., 2.2., and 2.4., areas in the recordings that contain no movement or entire recordings with no movement or almost no movement can be filtered out or ignored. The values for the window size, overlap, and threshold were validated in a grid search together with a manual inspection of the results. A higher threshold would have led to more filtering, possibly removing valid data.

Rules 1.1. and 1.2. are used to filter out short or defective recording files that were contained in the raw dataset. These very short recording files were sometimes accidentally created due to a loose contact or misplacement of the smartwatch in its charging bay, which in turn created very short, meaningless recordings. These recordings therefore did not contain any useful information and were discarded. Since the smartwatches were turned on before they were handed to the participants, rule 1.4. must be applied to delete non-study-related data, that was recorded before the participant received the smartwatch. Rule 2.1. is applied to ignore data from the initial hand wash that was conducted under supervision, to avoid the Hawthorne effect^44^. Finally, rules 2.3., 2.4., and 2.5. were used to clean implausible user input given by the participants. Sometimes, button presses were logged directly after starting a recording, indicating that the smartwatch had just been unplugged before the button was pressed. On some other occasions, the users labeled the same hand wash twice. We thus opted to ignore the labels at the beginning of a recording and all repetitions of labels that were too close to another handwashing label (10 *s*). This minimum temporal distance between two instances of handwashing was evaluated by manual inspection of some closely consecutive user annotations. The involved psychologists also confirmed it.

Overall, the filtering rules were created and used to make sure that the data contained in the final dataset was not larger than needed. Added to that they were applied so that the data is useful and informative and includes as few noisy user feedbacks as possible. We must, however, expect that a certain degree of possible user mistakes cannot be filtered from the labels. We still included the data that we marked to be filtered out, including idle regions. We did so to avoid permanently deleting it, in case other researchers may have a use for these data points. However, for researchers looking to train machine learning models with our dataset, we propose to make use of the “ignore” column to make training models and classification faster and easier. If the recording had gone exactly as planned, we would have expected to collect one file per day, leading to a total of 22 ⋅ 28 = 616 recording files, i.e., one file per day over 28 days for each of the 22 participants. However, we collected a total of 2121 recordings, out of which only 680 (32.1 %) were deleted during the data cleaning. The higher amount can be explained by participants connecting the smartwatches to the charger during the day. We chose a conservative approach and only deleted recordings of which we could be certain that they did not contain useful data. After applying the ignore rules, 58.6 % of the remaining dataset was marked to be ignored, due to the “no movement” rules. This is a relevant finding, indicating that some of the participants did not consistently wear the smartwatches as intended, or that they conducted long periods of almost no movement while wearing them. Since these periods with non-useful data would only prolong the computation duration, we decided to exclude them from our validation experiments.

For the sake of completeness, it is important to mention that we have chosen to disregard movement data occurring 5 minutes before and 1 minute following a labeled handwashing activity unless another activity immediately follows. This decision is based on a recent study conducted by Lønfeldt *et al*.^45^. We noticed instances where users occasionally forgot to promptly label a handwashing event by pressing the smartwatch button, only doing so minutes later. While we visually identified this pattern in the data, we cannot confirm it definitively, nor can we automatically filter or correct it. Therefore, to reduce potential confusion, we have decided not to consider this data for machine learning.

### Descriptive and Statistical Validation

**Fig. 4:**
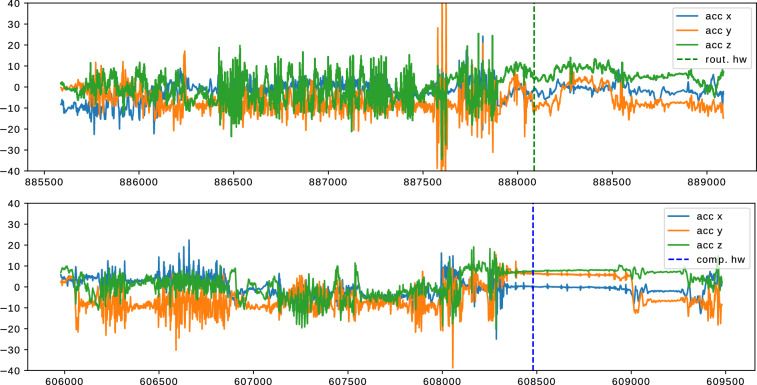
Examples of handwashing (top: routine, bottom: compulsive). Both examples are from the same recording of participant 03. The dotted vertical line indicates the position of the respective hand wash annotation placed by the user after washing. The plots show only the accelerometer data. A three-axis gyroscope is included in the recorded data as well. The x-axis is labeled with the respective count of samples since the recording started. One tick on the x-axis is equal to 500 samples, which is equal to 10 seconds of recording time.

From the records of 22 participants and the subsequent data cleansing (cf. Section: Data Cleansing), a dataset with 2600 hours of daily-life activities, including handwashing, was compiled. The recording duration of each participant averaged 118.2 ± 60.5 hours, taking into account variations in compliance. Inconsistencies in compliance could explain the significant differences between participants, with occasional situations of participants either forgetting to wear the smartwatch or experiencing technical issues, such as charging problems. The adjusted dataset includes a total of 2930 handwashing sessions, of which 1526 were categorized as compulsive by participants, while 1404 were identified as routine handwashing sessions. Figure 4 shows one example each of compulsive and routine handwashing.

#### Participant-Specific Insights

Given the findings from our initial data exploration, there are significant differences between participants in terms of compliance with the protocol and the quality of the data. Therefore, we analyzed individual participants in order to investigate these differences further.

**Fig. 5:**
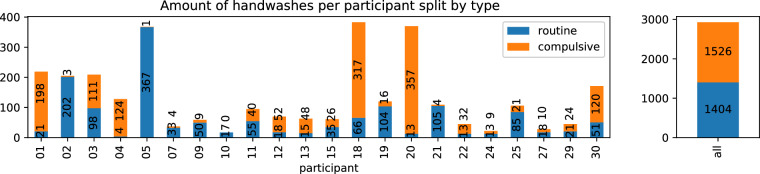
Amount of handwashing instances in the dataset, per participant (left) and overall (right), split by the user’s type annotation (compulsive - upper part of the bar, routine - lower part of the bar).

Figure 5 and Table 4 show how the different handwash types are distributed across the users. As with the motion data recorded in general, there are also large differences between the individual participants in the number of hand washes. For most participants, one would expect a high number of hand washes that are marked as compulsive. However, for some participants, a relatively high number of routine washes was recorded. For example, participant 05 recorded 367 routine hand washes compared to a single compulsive hand wash. The high amount of washes itself implies that most likely a large part of these washes was not routine washing. We conclude that participant 5 did not likely have enough insight to distinguish routine and compulsive handwashing objectively. The self-assessment-based label collection, therefore, leads to some user-induced errors in the resulting labels. The compulsive nature of handwashing was seemingly not always obvious to all participants.

**Table 4:** Hand washes by type per participant. The duration is the total duration of all recordings for the respective participant. The duration is very different between the participants, ranging from 26.88 *h* to 227.34 *h*, with a mean of 118.17 *h* ± 60.5 *h*. The average amount of compulsive washes is slightly higher than the average amount of routine washes. On average, each participant washed their hands 133.18 times (1.05 times per hour). Participants in bold print were seemingly most reliable in wearing the smartwatch regularly and creating plausible labels. Of 30 recruited participants, eight dropped out due to personal or technical reasons and are hence missing from the table.

Participant	Duration (h)	Hand Washes			Hand Washes / h		
		Compulsive	Routine	Total	Compulsive	Routine	Total
**01**	**161.91**	**198**	**21**	**219**	**1.22**	**0.13**	**1.35**
02	173.80	3	202	205	0.02	1.16	1.18
**03**	**131.58**	**111**	**98**	**209**	**0.84**	**0.74**	**1.59**
**04**	**139.09**	**124**	**4**	**128**	**0.89**	**0.03**	**0.92**
05	227.34	1	367	368	0.00	1.61	1.62
07	50.19	4	33	37	0.08	0.66	0.74
09	71.10	9	50	59	0.13	0.70	0.83
10	71.07	0	17	17	0.00	0.24	0.24
11	166.57	40	55	95	0.24	0.33	0.57
12	26.88	52	18	70	1.93	0.67	2.60
13	122.13	48	15	63	0.39	0.12	0.52
15	63.00	26	35	61	0.41	0.56	0.97
**18**	**185.67**	**317**	**66**	**383**	**1.71**	**0.36**	**2.06**
19	205.27	16	104	120	0.08	0.51	0.58
**20**	**205.64**	**357**	**13**	**370**	**1.74**	**0.06**	**1.80**
21	123.22	4	105	109	0.03	0.85	0.88
22	67.66	32	13	45	0.47	0.19	0.67
24	36.02	9	13	22	0.25	0.36	0.61
25	125.12	21	85	106	0.17	0.68	0.85
27	43.46	10	18	28	0.23	0.41	0.64
29	75.59	24	21	45	0.32	0.28	0.60
**30**	**127.49**	**120**	**51**	**171**	**0.94**	**0.40**	**1.34**
sum	2599.79	1526	1404	2930	—	—	—
mean	118.17	69.36	63.82	133.18	0.55	0.50	1.05

In the standardized psychological questionnaires, namely Y-BOCS^42^, some participants showed a relatively low score on insight and acceptance. The results of the questionnaires cannot be published per participant for ethical reasons. Still, by analyzing the questionnaires, we concluded that the labels entered by some participants could be trusted more than those from other participants. This also means that the quality and precision of the labels are not as high as they would have been for an in-lab study. In addition to sometimes noisy labels, some participants failed to wear the smartwatch for at least 6 hours per day. Overall, compliance and insight varied strongly between the participants. To react to this finding, we distinguished a subset of the seemingly most reliable and relevant participants with the clinical psychologists, consisting of participants [01, 03, 04, 18, 20, 30]. However, this subset does not necessarily include the best performers, as indicated by the results. These participants were selected according to carefully chosen criteria. The selection was mostly based on the answers to the psychological questionnaires, specifically on a score that indicated how much insight the participant showed about their symptoms (item 11 of Y-BOCS). We left out participants who lacked insight (score above one). We also only included participants with a total score on Y-BOCS of 14 and above in this subgroup. The value of 14 is slightly lower than the usual cut-off value of 16 for clinical relevance, but it allowed us to include one more participant in this subgroup. Although we especially focus on this subgroup in our further analysis, we also report technical validation results on the dataset as a whole. The distinction of the subgroup of likely more reliable participants was meant to help us train a better classifier for distinguishing routine from compulsive handwashing. However, the results for distinguishing handwashing from the NULL class are not different when only this subset is evaluated compared to all participants.

The resulting dataset contains around 31 *h* of handwashing and 2567 *h* of background data. Thus, the dataset is highly imbalanced, with a class imbalance of  ~ 99 % for the background data and  ~ 1 % for the handwashing activities. This impedes the training of classifiers, as the imbalance must be specifically taken into account and the results have to be treated and interpreted accordingly. However, the imbalance also reflects the realistic frequency of handwashing throughout the day compared to all other activities. For this reason, the class imbalance is to be expected according to the realistic prevalence of this activity. Thus, compared to other datasets recorded in the laboratory, our dataset can be used to obtain a more realistic estimate of the performance of a classifier designed to recognize hand-washing activities in a real-world setting.

#### Activity Insights

**Fig. 6:**
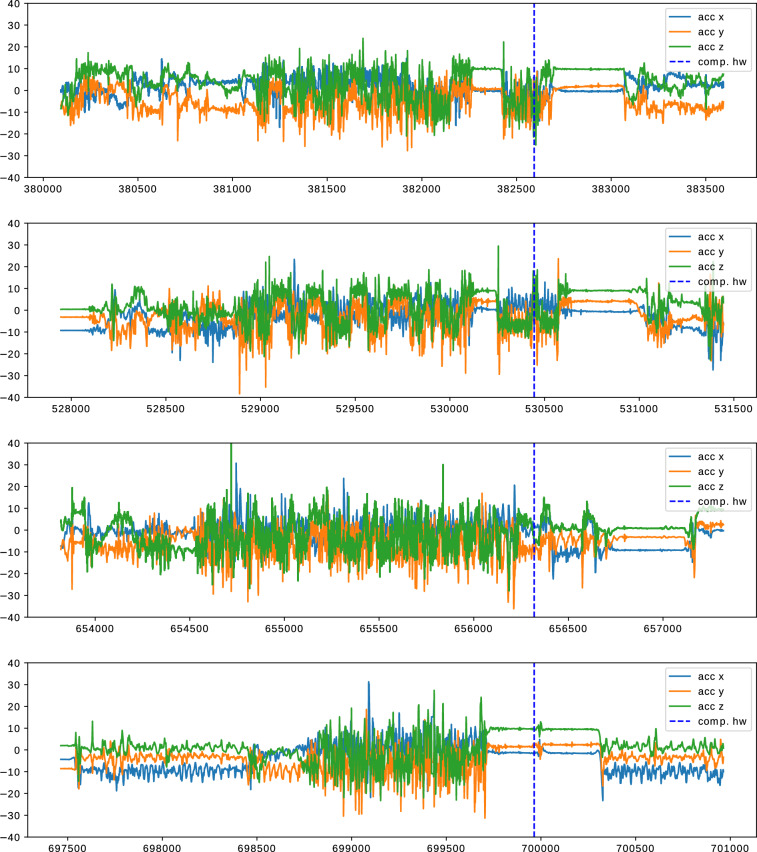
Accelerometer data for instances of handwashing. All hand washes are taken from the same recording of participant 20. The washes are of similar length and order of events. They start with a period of lower frequency movements and are followed by a period with higher magnitude and frequency. All washes in this figure were labeled as compulsive hand washes. The x-axis is labeled with the respective count of samples since the recording started, one tick on the x-axis is equal to 500 samples, which is equal to 10 seconds of recording time. The smartwatch was consistently worn on the same wrist.

**Fig. 7:**
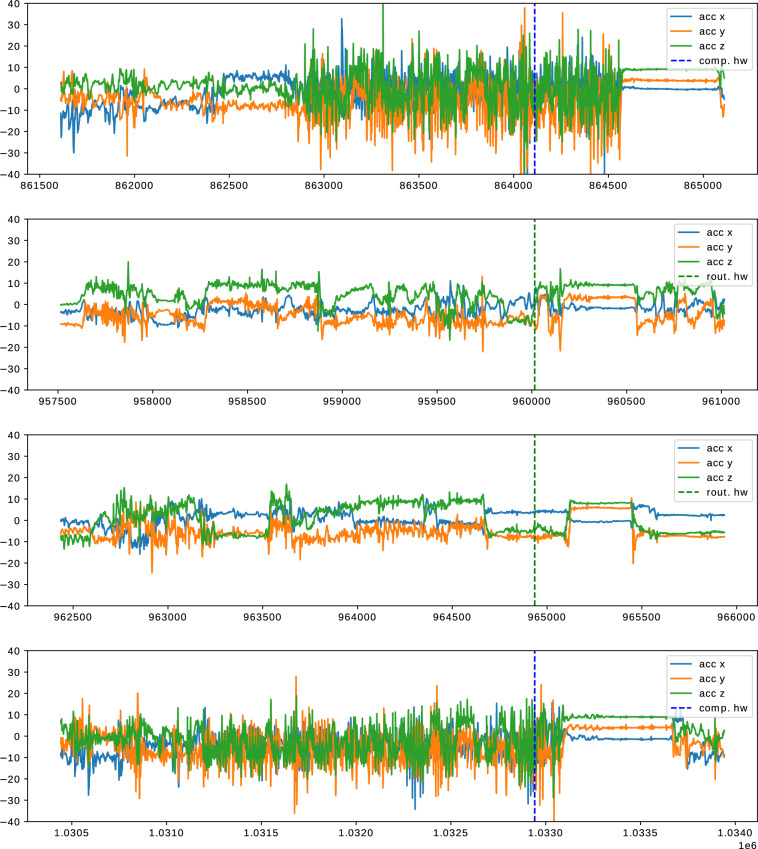
Accelerometer data for instances of handwashing. All hand washes are taken from the same recording of participant 20. The compulsive washes (top and bottom) contain movement with high frequency and magnitude, whilst the two routine hand washes (middle) show a lower frequency and magnitude. The x-axis is labeled with the respective count of samples since the recording started; one tick on the x-axis is equal to 500 samples, which is equal to 10 seconds of recording time. The smartwatch was consistently worn on the same wrist.

We visualized instances of compulsive and routine hand washing. Figure 6 shows examples of how self-similar a participant’s handwashing procedures can be. Figure 7 shows how different hand washes can be from other hand washes from the same person. For the participant displayed in the figures, the types of hand washing are visibly different, but this finding was not representative of all participants.

To break down the overall task of distinguishing between routine and compulsive handwashing, we initially conducted a statistical analysis. As with many experiments, we conducted the tests with the subgroup of six participants defined in the previous section (cf. Section: Participant-Specific Insights). By carrying out statistical tests, we wanted to get a sense of the nature of the different types of handwashing activities. We hypothesized that for training a simple classifier capable of discriminating between fairly similar activities, the difference in mean magnitude (definition for magnitude is presented in Section: Data Cleansing) should be statistically significant. Therefore, we stated the following hypotheses:

**Hypothesis**. *The mean acceleration magnitude differs significantly between compulsive and routine handwashing*.

**Null Hypothesis**. *The mean acceleration magnitude is the same for compulsive and routine handwashing*.

First, the Shapiro-Wilk test was used to check whether the data is normally distributed. It evaluates data from a sample with the NULL HYPOTHESIS that the data is normally distributed. A high p-value indicates that the data is normally distributed, and a low p-value indicates that it is not normally distributed. We have determined that the data is not normally distributed for all participants or that there is not enough data to make a clear and comparable statement. Therefore, as there is uncertainty about the overall normal distribution of the data, we opted for the Mann-Whitney U test. This non-parametric statistical test compares two independent samples to determine if they come from the same population. In our case, the outcome for all six participants did not allow rejecting the NULL HYPOTHESIS, suggesting the difference is not statistically significant. For both tests, we used the significance level *α* = 0.05. In summary, the statistical analysis emphasizes how difficult it is to distinguish different types of handwashing based on motion data alone. This finding is further supported by our proposed machine-learning approach.

##### Data Relabeling

The desired ground truth handwashing labels for the collected data would be sections consisting of start and end times for each instance of handwashing. Yet, due to the design of the study with the “one button press” after washing strategy, we did not initially collect exact times but only obtained a single timestamp indicating that a handwashing activity was performed previously. However, for training machine learning classifiers, we need a time span for the performed activities instead of individual data points. To obtain consistent labels corresponding to handwashing times, further data adaptation was required. Here we tried and implemented two different strategies to create continuous handwashing labels. In future work, other methods and new approaches for improving the labels could also be developed or tested using our dataset. An example of a solution could be data-driven or semi-supervised methods like SelfHAR by Tang *et al*.^46^.

##### Automatic Relabeling

The initial method aimed to automatically detect the beginning of handwashing activity and mark all subsequent samples as handwashing instances until a user label in the data indicated its end. We therefore used the first handwashing session, which was carried out in the lab. The videos of the participants washing their hands were manually inspected and compared with the data recorded on the smartwatches. In this way, we obtained data on the first hand wash, which consisted of one repetition of handwashing and its duration. To annotate every data point with a label, we calculated the average duration (38 *s*) of this single handwashing activity over all participants. We then manually inspected the handwashing data and decided on a reasonable 5 *s* offset between the end of the hand wash and the button press. We used this average duration of handwashing for all participants for whom we did not have an individual video for certain reasons. The preliminary labels were then set on the dataset with 38 *s* long intervals ending 5 *s* before the button press for the hand wash. However, for participants with existing lab video, we used the individual duration of the initial handwashing session to achieve a more individualized effect. These time spans range from 20 *s* being the shortest to 55 *s* being the longest. This method of setting dense labels is simple but has the disadvantage of not being entirely precise as to the exact timings of the start and end of the actual washing process. Each interval of handwashing was assigned either “compulsive”, or “routine”, depending on the user’s annotation. All samples outside of the handwashing intervals were assigned as “NULL”.

##### Manual Relabeling

The second, more complex and time-consuming approach consisted of manually adjusting the labels. As this approach is very extensive, we decided to only perform this as an exemplary approach for the subset of six participants.

**Fig. 8:**
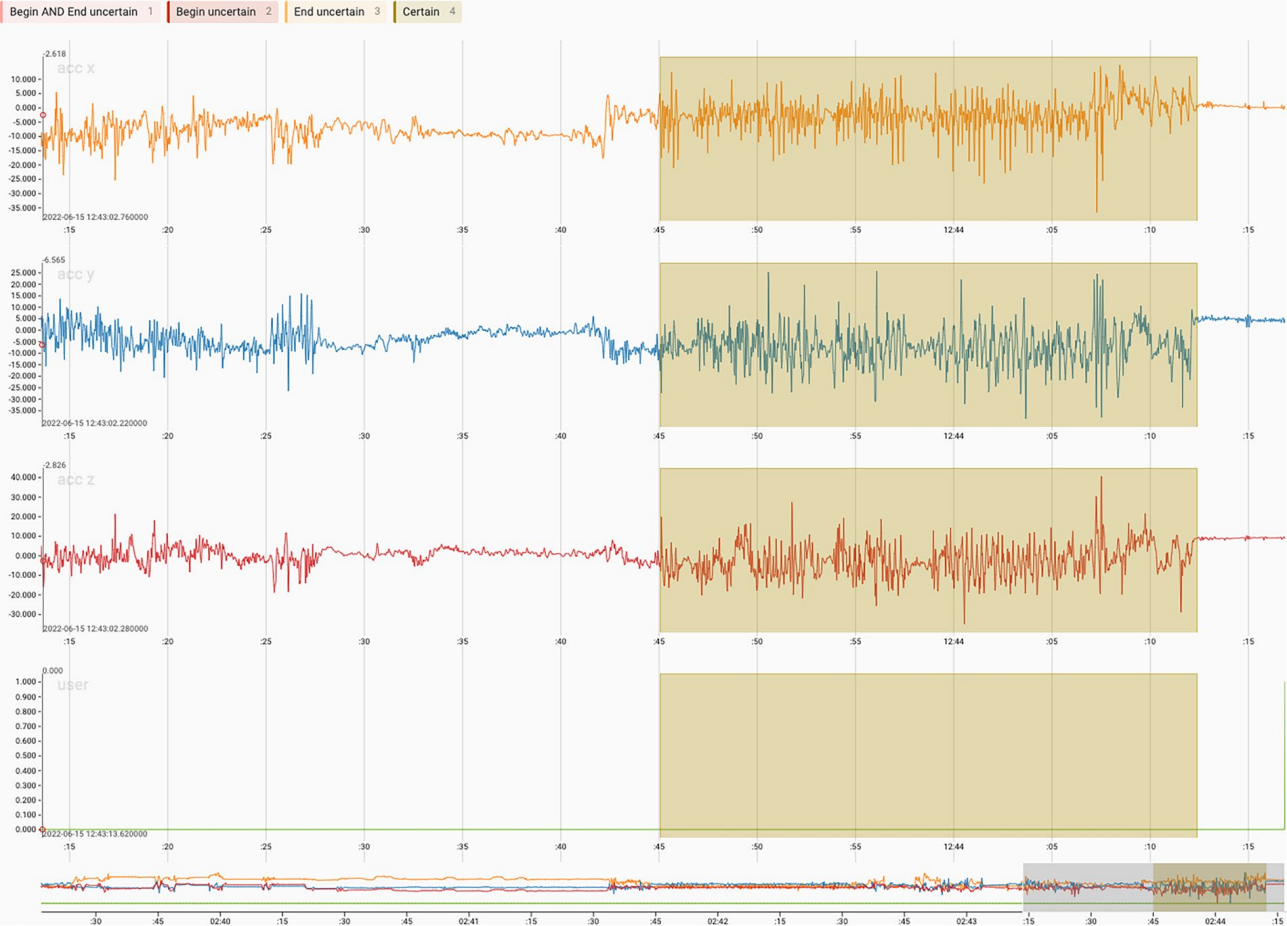
Example of a handwashing activity visualized in “Label Studio”. The top three charts display the data of the three axes of the accelerometer. The lower chart shows the original label (far right in green), which was set by the participant. The semi-transparent area is the label set manually by the annotator. The annotator could choose between four different label types, depending on certainty.

Setting manual labels requires knowledge about the topic and a technical background to be able to visually inspect motion data. In addition, a suitable tool must be selected that facilitates the work and visualizes the time-series signal data. We decided to use the open-source tool “Label Studio”^47^. The tool is easy to use and offers all functionalities needed to visualize IMU data and set manual labels. Figure 8 shows how a label was manually set in the web-based tool.

Since not every handwashing activity is clearly visible, the annotator could choose between four different label types (cf. Table 5), namely *Begin AND End uncertain* (if the activity start and end are difficult to identify), *Begin uncertain* (if only the end could be clearly determined), *End uncertain* (if only the beginning is identifiable) or *Certain* (if the activity is fully recognizable, as demonstrated in Fig. 8). Additionally, the annotator could also decide not to set a manual label at all, e.g., if there was no movement. This differentiation between label types enables us to subsequently conduct further analyses of the relabeling process. An analysis and evaluation of the approach with regard to the agreement of the annotators and a discussion of the limitations were also conducted^48^.

**Table 5:** Table listing the different label types available for annotators during manual relabeling of hand washes for the six participants of the subgroup.

Label Type	Identifier
No Label	0
Certain	1
Begin uncertain	2
End uncertain	3
Begin AND End uncertain	4

To mitigate personal biases in manual labeling, each participant underwent labeling by two distinct annotators. Consequently, across the six participants, we engaged four annotators to ensure every possible pair of annotators labeled each participant independently. For the following sections, when we describe that we used the manual relabeling approach as a data basis, we did not take the label type into account. We would rather treat all label types the same at this stage of the research. Nevertheless, since we have two independent annotations for each existing user label, we decided to use the union of both areas. Moreover, our pipeline offers the possibility to choose other approaches for dealing with the different annotations (e.g., using the intersection).

### Data Preparation

The data preparation step offers a variety of methods and flexible settings. We first filtered the IMU data using a Butterworth filter (*upper*_*threshold* = 18*Hz*, *lower*_*threshold* = 1*Hz*, *order* = 3) to deal with possible noise while preserving the original and significant patterns. Furthermore, we used a sliding window approach with 50% overlap, performing a majority vote for each window. The window size can be chosen individually. We tested different sizes from 1*s* to 15*s*. Even though the differences will be discussed in more detail later on, it can already be said here that a window size of 5*s* proved to be preferable, and we used this for many experiments.

#### Feature Engineering

For the machine learning classifiers, a dedicated feature engineering stage was introduced in the pipeline. To extract potentially relevant features, we used the Python library tsfresh^49^. The initial experiments relied on a minimal set of standard, use-case-independent features: maximum, minimum, mean, variance, standard deviation, median, and sum of values. Since these features capture only basic statistical properties, the set was extended to include additional frequency-domain features, based on the assumption that the frequency characteristics of handwashing might be particularly informative. The final feature set, covering both accelerometer and gyroscope data from all three axes, is listed in Table 6. All features were standardized by subtracting the mean and scaling to unit variance. Different feature selection strategies were applied, and during model training, the optimal subset was chosen by comparing three configurations: using all features, the 10 best features, or features selected via the select-from-model method. For the latter, a Random Forest classifier with a fixed random state (as in all experiments) was used, with the importance threshold set to the median. Subsequent analysis of feature importance revealed that certain sensor axes, such as the gyroscope x-axis and accelerometer z-axis, were consistently among the most relevant contributors across folds.

**Table 6:** The feature set to be used for the conventional machine learning experiments using the Python library tsfresh.

Feature	Parameters
mean	None
standard deviation	None
maximum	None
minimum	None
abs_energy	None
mean_abs_change	None
absolute_sum_of_changes	None
skewness	None
kurtosis	None
fft_aggregated	[{’aggtype’: ’centroid’}, {’aggtype’: ’variance’}, {’aggtype’: ’skew’}, {’aggtype’: ’kurtosis’}]
fourier_entropy	[{’bins’: 2}, {’bins: 10}, {’bins’: 100}]

#### Data Resampling

Since we are dealing with real-world data and thus high class imbalances (99% vs. 1%), we also introduced the option of resampling the original data to achieve a more balanced distribution. With regard to the literature, we decided to try both under- and oversampling strategies as well as a mixture of both. For oversampling the data we tried out the synthetic minority over-sampling technique (SMOTE)^50^, for undersampling we used a random undersampler, and for the mixture, we tried out two variations of SMOTE (SMOTE and Tomek Links^51^, and SMOTE and Edited Nearest Neighbours^52^). For the mixed strategies, we opted to downsample only the majority class. Surprisingly, none of the strategies tested stood out noticeably from the others. Sampling the majority class down has even delivered the best results in most cases, which is advantageous compared to the computational time when the extremely small class has to be sampled up.

### Machine Learning

To capture a variety of simpler machine learning approaches, we utilized state-of-the-art conventional machine learning models to present baseline classifiers for the OCDetect dataset. We primarily focused on distinguishing handwashing from the NULL class, treating both types of handwashing as the positive class and the background data as the negative class. Although the statistical tests showed that the different types of handwashing are not significantly different, we also trained and evaluated the same classifiers and classification pipeline on the task of distinguishing between routine and compulsive handwashing. In this task, routine handwashing serves as the negative class, while compulsive handwashing is the positive class.

#### Classifiers

For classical machine learning, we concentrated on three well-established classifiers: Random Forest (RF), Gradient Boosting Classifier (GBC), and Logistic Regression (LR). To determine the optimal model configurations, we employed a small grid search (cf. Table 7) and, overall, a nested leave-one-subject-out cross-validation. The latter refers to the fact that we divide the training, test, and validation sets at the participant level to reduce bias, avoid over-fitting, and gain a realistic approximation of the model’s performance on unseen participants.

**Table 7:** Parameters and values for the grid search cross-validation for the conventional models.

Model	Parameter	Grid Search Values
Random Forest Classifier	n_estimators	100
	criterion	entropy, gini
	max_depth	10
	max_features	sqrt, log2
Gradient Boosting Classifier	loss	log_loss, exponential
	learning_rate	0.01, 0.3
	n_estimators	100
	max_depth	10
	max_features	sqrt, log2
Logistic Regression	solver	saga
	max_iter	5000
	penalty	elasticnet
	l1_ratio	0.0, 0.5, 1.0
	C	0.1, 5.0

We created and evaluated these classical machine learning classifiers to demonstrate the feasibility of detecting handwashing in our dataset. These classifiers do not necessarily represent the state-of-the-art in human activity recognition (HAR) using sensor data; however, they have been widely employed in prior publications showing good results. We expect that better performance can be achieved by optimizing the pipeline and/or using other models.

Convolutional, recurrent, and transformer-based networks have demonstrated their effectiveness in temporal activity recognition tasks in the past^53,54^. Therefore, to incorporate a more state-of-the-art classifier, we additionally evaluated a deep learning model. Initially, we experimented with the well-established DeepConvLSTM^24^, since Convolutional Neural Network (CNN)-Long Short-Term Memory (LSTM) architectures continue to deliver competitive performance in inertial HAR. However, this is likely due to the extremely high class imbalance in the training data, particularly the scarcity of positive class samples and the overall difficulty of the task. As a result, training the DeepConvLSTM was unstable and failed to converge to effective classification weights. After identifying this issue with training stability, we adopted a more advanced approach by employing HARNet, a wrist IMU model pre-trained in a self-supervised manner on 700,000 person-days of wrist IMU data^55^. The HARNet model is a CNN with residual connections, for which pre-trained weights are freely available. Since our own dataset proved to be challenging when training models from scratch, the pre-trained HARNet was used to provide embeddings for the accelerometer and gyroscope data. We obtained a 2048-value accelerometer and gyroscope embedding vector for each sample window. In addition to these embeddings, we trained a fully connected 2-layer classification head ([1024, 2] neurons) using a class-weighted focal loss. The weighted focal loss function assigns higher weights to difficult samples and was chosen to account for the natural class imbalance in our dataset. The focal loss function performed significantly better than the binary cross-entropy function.

Applying more extensively optimized deep learning methods, which would require a thorough exploration of architectures and hyperparameters, was beyond the scope of this dataset contribution. In general, deep learning classifiers tend to outperform conventional approaches when sufficient training data is available, although this advantage may diminish in data-scarce scenarios.

#### Post-Processing

To fine-tune our classification, we also introduced an optional post-processing step into the pipeline. This idea was based on the fact that the sliding window approach aims to classify each window independently, without considering the added temporal information from the surrounding windows. Since one occurrence of handwashing typically spans multiple windows, it makes sense to accumulate the predictions from multiple windows as well. We can utilize the temporal context of neighboring windows by post-processing the initial ’raw’ predictions of the classifier using a moving average filter. We used the moving average filter to average the predictions across multiple windows. This can also be seen as a smoothing of the signal. Mathematically, the filter can be viewed as a convolution with a box kernel. To calculate the post-processed value for a prediction vector $\overrightarrow{p}\in {\{0,1\}}^{n}$ and kernel size *k*, we slide over the prediction vector and calculate the post-processed prediction $\overrightarrow{pp}$ for each index *i*: $p{p}_{i}=\frac{1}{k}{\sum }_{j=i}^{k+i}{p}_{j}$. We applied this post-processing step with a kernel size of *k* = 5 to the initial predictions of each model. Depending on the window size, this means that sensor data of 15 *s* to 45 *s* influences the post-processed prediction at each index. On average, the post-processing increased the resulting F1 score by 0.03. However, for participant 20, a maximum improvement of the F1 score of 0.11 was reached. This shows that improved performance can be achieved using this or a similar post-processing step, especially if the underlying model is already performing well. We also experimented by replacing the box-kernel with a Gaussian kernel, but this change decreased the achieved improvement. The post-processing step is applicable to the detection of handwashing in the real world, but it requires a minor modification since we convolve over predictions that lie in the future. However, if we need to detect handwashing in an online paradigm, we can simply average over the last *k* model outputs to achieve a similar effect. To extend this post-processing approach, future research could also optimize for the kernel size or use a different kernel, such as a learned kernel, to better weigh the predictions for windows at different distances.

### Evaluation

When dealing with real-world data and given the nature of our use case, which involves highly imbalanced data, a suitable evaluation metric must be chosen to interpret the results accurately. Currently, the predominant method for this remains the utilization of the F1 score. The F1 score takes into account both precision and recall, making it robust in scenarios where classes are unevenly distributed, whereas accuracy can be misleading when only the dominant class is predicted. The F1 score is defined as follows:

$${F}_{1}=\frac{2\cdot \,precision\cdot recall}{precision+recall}$$

Nevertheless, interpreting the F1 score without additional information remains challenging. Therefore, we also present the chance level represented by a dummy classifier. To obtain the best-performing chance level classifier, different random and fixed-value dummy classifier strategies were evaluated using their F1 score. The best-performing dummy classifier in our case was the one always predicting “hand wash” (minority class / positive class). Based on this chance-level classifier, we can demonstrate the extent to which our models can deliver better predictions. In addition, we also examined other metrics, such as the receiver operating characteristic (ROC) and the precision-recall (PR)–curve, along with their respective area under the curve (AUC) values. Finally, by examining both the test-participant-specific and the overall confusion matrices, we could gain a deeper understanding and more accurate estimates.

In the context of (compulsive) handwashing detection, both Type I and Type II errors have significant implications. A Type I error (false positive) may incorrectly flag a non-wash or a normal handwashing event as compulsive, potentially leading to unnecessary concern, intervention, noisy data records, or annoying the user with unwanted interactions. Similarly, many Type II errors (false negatives) could limit the system’s perceived reliability and mislead the treating psychologists. Minimizing, yet also balancing, both types of errors is therefore crucial to ensure both sensitivity and specificity in the detection system. The choice of a classification threshold in a clinical setting must reflect the priorities of minimizing harm while maximizing detection accuracy. This choice can thus only be made in conjunction with domain experts from the field of psychology.

#### Classification Results

An overview of the initial results for the setup described above is presented in Table 8. To compare the received F1 scores to the chance level, the table also includes the results for a chance-level classifier. We utilized the F1 score to determine the optimal threshold for the decision boundary and to identify the best parameter values for the models via a comprehensive grid search. We found that a window size of 5 *s* is the most promising one after testing smaller and larger windows. Finally, we estimated the performance of each model on the held-out participant. To ensure the reproducibility of our results, we adopted a consistent seed value for all random number generators in our experiments. Since we achieved slightly better results when using our manual relabeling approach, we present those results for the subgroup here. Overall, the decision-tree-based models, RF and GBC, perform slightly better than the LR.

**Table 8:** Overview of the results of the first experiments for the machine learning models for the binary classification task to distinguish hand washing in general from all other activities. The results were created on the subset of the six participants with manually relabeled data. The table shows the individual F1 scores with and without (indicated as w/o) post-processing (abbreviated as pp). All results are produced for a window size of 5*s*. We present the results for the best configurations for a Random Forest (RF), Gradient Boosting Classifier (GBC), Logistic Regression (LR), and Deep Learning (DL). The values refer to the optimal model of the leave-one-subject-out cross-validation tested on the respective subject. For a better evaluation of the results, an additional chance level classifier for a window size of 5 seconds is given as a baseline. The chance level is determined by the highest-performing dummy classifier, which always predicts the positive class.

Model	LOSO-trained, tested on Participant													
	01		03		04		18		20		30		avg	std
	w/o pp	with pp	w/o pp	with pp	w/o pp	with pp	w/o pp	with pp	w/o pp	with pp	w/o pp	with pp		
RF	0.12	0.13	0.34	0.40	0.22	0.25	0.32	0.33	0.65	0.76	0.12	0.12	0.31	0.21
GBC	0.12	0.12	0.37	0.40	0.24	0.28	0.32	0.34	0.71	0.77	0.12	0.13	0.33	0.22
LR	0.10	0.10	0.30	0.34	0.19	0.21	0.35	0.36	0.72	0.75	0.11	0.11	0.30	0.23
DL	0.11	0.13	0.11	0.15	0.10	0.15	0.28	0.36	0.45	0.65	0.09	0.11	0.26	0.21
Chance	0.02		0.03		0.02		0.03		0.04		0.04		0.03	

The classical methods all outperform the Deep Learning (DL) models by a small margin. With the recent success of deep neural networks in HAR, one might expect the DL model to perform best. In our case, however, the classical methods achieved higher performance given the window size and dataset distribution. For our short, 5-second-long windows, classical methods likely perform better because the handcrafted features already summarize the relevant temporal patterns, enabling well-regularized classical models to exploit these compact, low-variance representations more effectively than high-capacity DL or LSTM based architectures.

The tree-based models performed best in our early-stage analyses for several reasons. The amount of positive-class data available per participant is limited, and compulsive handwashing shows considerable variation between individuals. Tree-based methods handle such heterogeneous data well and can model nonlinear patterns without requiring large datasets. The data also shows notable class imbalance, and tree ensembles tend to remain robust in these scenarios, especially when combined with resampling techniques^56^. Additionally, sensor signals often contain noise and occasional irregularities, and tree-based models tend to be less sensitive to these issues than deep neural networks. Deep learning approaches, by contrast, typically need larger and more diverse datasets to learn stable and generalizable representations. To benefit from pre-trained deep models in this setting, we would likely require substantially more patient data and a better understanding of how compulsive handwashing manifests in OCD. With an easier class balance, longer windows of sensor data, or other means of achieving a more stable training process, we would still expect that CNN-LSTM architectures outperform classic models because they are usually better at incorporating long-term trends and dependencies within the time-series data.

Overall, the comparatively high standard deviation indicates that the classifier behaves differently for each participant. This illustrates the uniqueness of everyday behavior and the challenge of developing a classifier that works reliably for most people.

We also calculated the balanced accuracy, recall, precision, Matthews Correlation Coefficient (MCC), and F1 scores for all participants using the automatic relabeling approach (cf. Section: Automatic Relabeling), a window size of 5 s for a GBC with a fixed parameter setup, and the proposed post-processing step. The GBC was selected as it outperformed the other models for the participant subgroup. The table listing the results is provided in Table 9. Furthermore, for a comprehensive overview of feature relevance, a table with the top features across all folds is also shown in Table 10.

**Table 9:** Overview of the results for all participants with automatic relabeling and a window size of 5 s. The table shows Balanced Accuracy, Recall, Precision, MCC, and F1 Score after post-processing for a GBC with a fixed parameter setup (loss=exponential, learning_rate=0.01, n_estimators=100, max_depth=10, max_features=sqrt). The values represent the results for the classifier for each test participant in the leave-one-subject-out cross-validation.

Test Participant	Balanced Accuracy	Recall	Precision	MCC	F1 Score
01	0.54	0.08	0.09	0.07	0.08
02	0.73	0.47	0.62	0.53	0.53
03	0.65	0.30	0.62	0.43	0.40
04	0.59	0.18	0.54	0.31	0.27
05	0.55	0.10	0.16	0.12	0.13
07	0.67	0.34	0.69	0.48	0.46
09	0.54	0.09	0.30	0.16	0.14
10	0.51	0.02	0.03	0.02	0.02
11	0.61	0.21	0.41	0.30	0.28
12	0.67	0.36	0.43	0.38	0.39
13	0.70	0.40	0.46	0.43	0.43
15	0.53	0.06	0.30	0.13	0.10
18	0.65	0.30	0.60	0.41	0.40
19	0.53	0.07	0.25	0.13	0.11
20	0.76	0.53	0.79	0.64	0.63
21	0.66	0.33	0.70	0.48	0.45
22	0.57	0.15	0.59	0.29	0.24
24	0.61	0.23	0.41	0.30	0.29
25	0.56	0.12	0.29	0.19	0.17
27	0.52	0.05	0.18	0.09	0.07
29	0.51	0.03	0.19	0.07	0.05
30	0.53	0.07	0.19	0.11	0.11
**avg**	0.61	0.23	0.43	0.29	0.26
**std**	0.07	0.16	0.21	0.16	0.17

**Table 10:** Top 15 most frequent features across all folds (22 participants) based on top-10 feature importances. The features are derived from the same GBC results reported in Table 9, obtained with automatic relabeling and a window size of 5 s. Features originate from both accelerometer and gyroscope data across all three axes and are ranked by their frequency of occurrence in the top-10 importances across folds.

Feature Name	Count in Top 10	% of Folds	Avg. Rank	Mean Importance
gyro x__absolute_sum_of_changes	22	100.00	1.95	0.23
acc z__absolute_sum_of_changes	22	100.00	2.14	0.20
acc z__mean_abs_change	22	100.00	2.86	0.14
gyro x__mean_abs_change	22	100.00	3.05	0.13
gyro x__fft_aggregated__aggtype_kurtosis	22	100.00	5.05	0.04
acc x__fft_aggregated__aggtype_centroid	21	95.45	7.10	0.02
acc z__fft_aggregated__aggtype_skew	20	90.91	6.95	0.02
acc y__fft_aggregated__aggtype_centroid	20	90.91	7.45	0.02
gyro x__abs_energy	11	50.00	9.09	0.01
acc z__fft_aggregated__aggtype_centroid	10	45.45	8.90	0.01
acc z__maximum	7	31.82	8.43	0.01
gyro x__standard_deviation	6	27.27	8.83	0.01
acc x__standard_deviation	4	18.18	10.00	0.01
acc x__fft_aggregated__aggtype_kurtosis	3	13.64	9.33	0.01
acc z__abs_energy	3	13.64	9.33	0.01

**Fig. 9:**
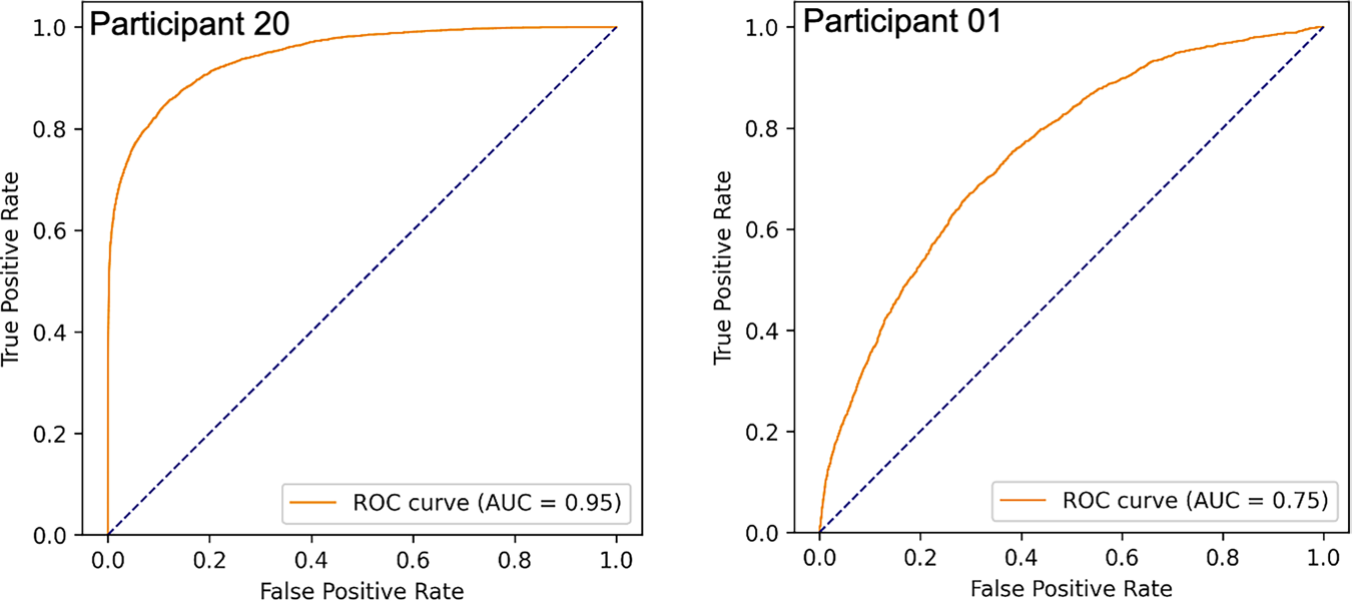
ROC curves for the GBC after post-processing, showing an example with the highest F1 score and AUC (participant 20, left figure) and one with a very low F1 score (participant 01, right figure).

This analysis shows that certain features, such as gyroscope x-axis and accelerometer z-axis values, are consistently among the most relevant contributors across folds. It is not meaningful to present an overall ROC curve in this context, as the class distributions, decision thresholds, and operating points vary across participants, making a global ROC representation potentially misleading. However, for completeness, we provide the ROC curves with their respective AUC values for the model achieving the highest F1 score (testing on participant 20 with AUC=0.95) and for one of the lowest-scoring cases (participant 1 with AUC=0.75) in Fig. 9.

For the task of distinguishing routine handwashing from compulsive handwashing, no classifier could significantly outperform the chance level of the dummy classifiers. We thus do not explicitly report the results from this computation. It should be noted that for this task, the class balance varies a lot between the different participants. Many participants have mostly conducted one type of hand wash, either routine or compulsive. While the F1 score is the perfect metric for evaluating the task of spotting a few hand washes in a dataset with a large NULL class, the F1 score is not the most suitable metric for evaluating classification problems in which the relevant class (compulsive) is larger than the negative class (routine). Rather, a balanced accuracy score, ROC-AUC, PR-AUC, MCC or similar metrics could be employed. The MCC, in contrast to the F1 score, takes into account true positives, true negatives, false positives, and false negatives, providing a balanced measure of classification performance that is robust to class imbalances.

## Usage Notes

In general, the provided dataset can be used for handwashing detection in OCD related and unrelated settings. Depending on the research question, the dataset could also be used to augment other HAR tasks. Large parts of our dataset are unlabeled background data, which could be added to lab-recorded data to achieve higher class variety and robustness against background data. Its large amount of realistic everyday activity data could also lend itself well to unsupervised or self-supervised pre-text training. The user-annotated *compulsive* label can be used to analyze differences in the contained handwashing data. For example, the length of the hand washes or their movement energy could be compared between different types of hand washes.

In order to repeat the steps described in this work, one should download both the code and the dataset from the hyperlinks provided in Section: Data Records and Section: Code Availability. The downloads must be extracted and the value *data_folder* in *misc/config/config.yaml* must be adapted to include the correct relative paths between the code folder and the dataset files. Added to that, a Conda environment should be created and activated using Python 3.10. The packages in *requirements.txt* must then be installed (*pip install -r requirements.txt*). Afterward, the experiments can be run with the command *python main.py*. The executed Python scripts will then generate the results and save them to the directories specified in *misc/config/config.yaml*. To change the parameters of the execution, values in *misc/config/settings.yaml* can be adjusted.

## Data Availability

The dataset is openly available on Zenodo^43^, and can be downloaded using this url: https://zenodo.org/records/13924901. Refer to the Data Records Section for more details.

## Code availability

The code used to preprocess, annotate, and analyze the dataset can be found at https://github.com/OCDetect/OCDetect-pipeline. The repository includes a file called *ReadMe.md*, which contains step-by-step instructions on how to execute the pipeline to repeat our processing and experiments.
